# Cardiovascular Diseases in Natural Disasters; a Systematic Review

**DOI:** 10.22037/aaem.v9i1.1208

**Published:** 2021-05-04

**Authors:** Javad Babaie, Yousef Pashaei asl, Bahman Naghipour, Gholamreza Faridaalaee

**Affiliations:** 1 Department of Health Policy& Management, Tabriz University of Medical Sciences, Tabriz, Iran.; 2 Tabriz Health Services Management Research Center,Tabriz University of Medical Sciences, Tabriz, Iran.; 3 Iranian Center of Excellence in Health Management, Tabriz University of Medical Sciences, Tabriz, Iran.; 4 Department of Health Services Management, School of Health Management and information Sciences, Iran University of Medical Sciences, Tehran, Iran.; 5 Department of Anaesthesiology and Intensive Care, Tabriz University of Medical Sciences, Tabriz, Iran.; 6 Emergency Medicine Research Team, Faculty of Medicine, Tabriz University of Medical Sciences, Tabriz, Iran.; 7 Department of Emergency Medicine, Maragheh University of Medical Sciences, Maragheh, Iran.; 8 Disaster Research Team, Tabriz University of Medical Sciences, Tabriz, Iran.

**Keywords:** Natural disasters, Earthquakes, Floods, Cardiovascular Diseases, Hypertension, Acute Coronary Syndrome

## Abstract

**Introduction:**

As a result of destruction and lack of access to vital infrastructures and mental stress, disasters intensify cardiovascular diseases (CVDs) and hence management of CVDs becomes more challenging. The aim of this study is investigating incidence and prevalence of CVDs, morbidity and mortality of CVDs, treatment and management of CVDs at the time of natural disasters.

**Methods:**

In the present systematic review, the articles published in English language until 28. 11. 2020, which studied CVDs in natural disasters were included. The inclusion criteria were CVDs such as myocardial infarction (MI), acute coronary syndrome (ACS), hypertension (HTN), pulmonary edema, and heart failure (HF) in natural disasters such as earthquake, flood, storm, hurricane, cyclone, typhoon, and tornado.

**Result:**

The search led to accessing 4426 non-duplicate records. Finally, the data of 104 articles were included in quality appraisal. We managed to find 4, 21 and 79 full text articles, which considered cardiovascular diseases at the time of flood, storm, and earthquake, respectively.

**Conclusion:**

Prevalence of CVD increases after disasters. Lack of access to medication or lack of medication adjustment, losing home blood pressure monitor as a result of destruction and physical and mental stress after disasters are of the most significant challenges of controlling and managing CVDs. By means of quick establishment of health clinics, quick access to appropriate diagnosis and treatment, providing and access to medication, self-management, and self-care incentives along with appropriate medication and non-medication measures to control stress, we can better manage and control cardiovascular diseases, particularly hypertension.

## Introduction

Over recent years, the number of disasters and their costs has been increasing and is 6 times higher compared with the first half of the last century (1-3). For instance, about 324 disasters with 141 million casualties occurred only in 2014 (4). In addition to destroying homes, roads, drinking water system, electricity and gas system, and causing other economic damages, disasters lead to an increase in the incidence of communicable diseases, non-communicable diseases (NCDs), and trauma (5). NCDs were the leading cause of mortality and morbidity in the world over the last century and their incidence and prevalence have an increasing trend. It is expected that incidence and prevalence of NCDs increase at the time of disasters and the people present in the disaster area be more vulnerable to NCDs (6, 7). 

Cardiovascular diseases (CVDs) is the main category of NCDs whose incidence and prevalence have an increasing trend due to changing life style and aging population (8-10). NCDs leads to 40 million deaths in the world each year and like other NCDs, incidence and prevalence of CVDs increases after disasters (9). As a result of destruction and lack of access to vital infrastructures such as homes, health centers, medication and also causing physical and mental stress, disasters intensify CVDs and hence management of cardiovascular diseases faces a fundamental challenge (10-13). The aim of this systematic review study is investigation of incidence and prevalence of CVDs, morbidity and mortality of CVDs, and treatment and management of CVDs, at the time of natural disasters. 

## Methods

This is a systematic review based on PRISMA protocol. In this study, PICO is defined as: P, which stands for problem or population, is individuals with cardiovascular diseases, (I) is natural disasters, (C) comparing normal situation, and the (O) outcome is prevalence, treatment, and management of CVDs. 

**Eligibility Criteria**

In the present study, the articles published in English language until 28. 11. 2020, which studied CVDs in natural disasters were included. The inclusion criterion was study of CVDs such as myocardial infarction (MI), acute coronary syndrome (ACS), hypertension (HTN), arrhythmia such as atrial fibrillation (AF), ventricular tachycardia (VT), ventricular fibrillation (VF), and paroxysmal supraventricular tachycardia (PSVT), pulmonary edema, and heart failure (HF) in natural disasters such as earthquake, flood, storm, hurricane, cyclone, typhoon, and tornado. The articles published in the form of abstract as a poster, conference proceeding, commentary, editorial, and case report were excluded. In this study, volcano and climate changes were not included. Similarly, man-made disasters were excluded and not reviewed. 

**Search Strategy**

In order to achieve the purpose of the present study, search items and their related key terms were selected by means of using MeSH and EMtree databases, consulting with expert specialists, searching the titles and abstracts of the related articles under supervision of a specialist and researcher in emergency medicine and a health management in disasters Ph.D. An extensive search in electronic databases including Medline, Web of Science, Embase, and Scopus until 28. 11. 2020 was done. Search strategy in Medline database is presented in table 1. 

**Study Selection and Data Collection Process and Outcome Appraisal**

 In this study, all articles published in English language, which studied CVDs in natural disasters, were included. Screening of the articles was done based on inclusion and exclusion criteria. First, abstracts of the articles were read by two independent researchers. Then, after selecting the eligible articles, full texts of the articles were evaluated. Afterwards, the full text was considered in accordance with inclusion and exclusion criteria and eligible articles were selected. Summarizing the articles and recording the data in the checklist along with final quality control was performed by two independent individuals. Any discrepancy in views was resolved through discussion between two parties or by means of consulting a third researcher. The articles were summarized using a checklist, which has been designed based on PRISMA statement (14). In this systematic review, outcome appraisal was prevalence, treatment, and management of CVDs in natural disasters. Data related to first author and year of publication, being peer reviewed, obtaining ethical or publication committee approval, definition of the outcome, expression of exclusion criteria, presence of a control group, and expression of statistical method were extracted. 

**Statistical analysis**

Data analyses were done in a descriptive way. All the articles were summarized and categorized based on the considered variables. 

Ethics

Since systematic review studies consider the previously published studies and the research is not directly done on human or animal, there is no need for ethical approval. 

## Results

**Study Selection and** **Study Characteristics**

The search led to 4426 non-duplicate records. 4,199 abstracts were excluded as they were not related to the purpose of our study. Also, 115 studies were case report, letter to editor or Correspondence, review articles, abstracts presented at the conferences and non-English, all of which were excluded from the study. 112 article abstracts were eligible and hence necessary measures to provide their full text were taken. Also, six full text articles were studied but since they did not meet our criteria, they were excluded. We were not able to the find full text of two articles. They were not even accessible in the journal archive. Finally, the data of 104 articles were included in quality control appraisal. We managed to find 4, 21 and 79 full text articles, which considered cardiovascular diseases at the time of flood, storm, and earthquake, respectively. 

**Figure 1:**
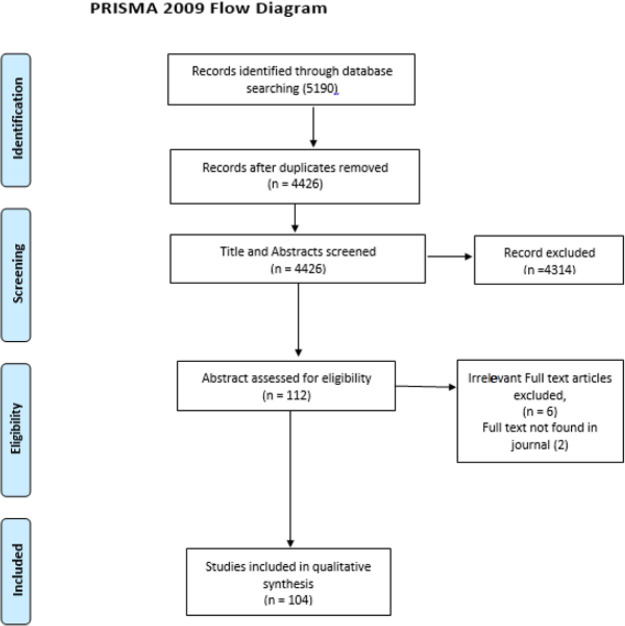
PRISMA flow diagram

The selection process and PRISMA diagram are shown in Figure 1. Due to the variety of our included articles, based on natural disasters, we grouped the included articles into 3 categories including storm (hurricane, typhoon, Cyclone and Tornadoes), flood, and Earthquake.

**Quality control of study and risk of Bias**

The included articles were qualitatively considered. The qualitative review results of flood, storm (hurricane, cyclone, typhoon, and tornadoes), and earthquake are presented in Table 2, 3, and 4, respectively. 

**CVDs in Flooded Areas**

Prevalence of CVDs increases after flood. Diseases like AF, PSVT, ACS, severe CHF, cardiopulmonary arrest, and AMI undergo a remarkable increase in the first week and then decrease. The second wave of increase in the number of CVDs is also observed in the 7^th^ week (15). Existence of negative experiences such as loss of property, physical work, financial problems, alcohol use, and perceived distress in the long run can lead to hypertension (16). Nevertheless, some studies indicated that despite the increasing prevalence of CVDs after flood, such an increase is not statistically significant (17) and when confounding factors are excluded from the study, increase in the prevalence of CVD is not observed (18). 

**CVDs in Storm area (hurricane, Typhoon, Cyclone and Tornado)**

Prevalence of CVDs including HTN, AMI, and fatality caused by CVDs increase after hurricane (19-24). In the areas extremely affected by the hurricane, the rate of CVDs, particularly HTN, is high (23, 25). Unemployment, drug abuse, smoking, temporary housing life, and lack of health insurance are among the risk factors of increase in the prevalence of CVDs, particularly AMI (20-22, 26). After hurricane, CVDs obviously increase in women over 45 years of age; however, in the 6-month follow-up, no increase is observed (27). In terms of circadian and septadian rhythms, studies indicated that within 3 years, and in some studies within 6-10 years, after hurricane, the rate of CVDs increased only on the evenings and weekends. However, on the following morning and the first day of the week, a considerable decrease is observed in the prevalence of AMI (2, 21, 22). Since tornado has a small volume and does not take more than some minutes, it does not cause increase in cardiovascular diseases (28).

At the time of evacuation, some patients forget to take their medication out of their home and some of them run out of medication or cannot obtain them and lack of medication makes them unable to control their HTN and hence uncontrolled HTN increases (29, 30). The issue is so prevalent and 48.4% of those who are taken to shelters lack medication, most of whom are male and have no health insurance (31). Also, about 10% of the patients, who are taken to shelter, have chest pain and require emergent treatment (31). After hurricane, adherence to medication regimen decreases, particularly in individuals over 65 years of age and non-whites, (26), which causes more uncontrolled HTN in these individuals in comparison with those who have higher adherence to antihypertensive drugs (29). However, after one year and in the second year after hurricane, adherence to medication returns to its previous state (32). The other factor leading to higher and uncontrolled HTN is stress (33); particularly in the elderly, it causes an increase in CVDs and lack of controlled HTN (34, 35). Reasons for such stress factors include lower capability of coping with disaster, more damage to living place, stress of living after hurricane, increase in separation from friends and family, fewer visits to friends and family, loss of property and relatives (33). Medication request rate is higher in patients with CVDs in comparison with other diseases. Although only 11% of the complaints belong to the patients with CVDs, 52% of the requests for medication are for CVDs (36). Also, 55.6% of the individuals, who live in shelter, suffer from chronic diseases like HTN, diabetes, hypercholesterolemia, pulmonary diseases, and mental disorders (31). The amounts of medication required for chronic diseases and CVDs make up a high percentage of the medication required during hurricane, which are 68% and 39%, respectively (37). Temporary reduction in access to health care centers leads to a decrease in the number of patients referring for primary care after reopening of the centers and as a result more uncontrolled HTN can be observed (28). 

To improve the quality of health care services, the following points are recommended (30): 1. Having electronic health records, which enables the treatment staff to have access to the history of patients, prescribe the previous drugs of the patient appropriately and quickly, and to better control chronic diseases upon emergencies. 2. Electronic health records backup. 3. Appropriate storage of medications 4. All members of healthcare provider team should be aware of the plan and their own roles, and 5. For times when telephone and internet disconnect, there should be a backup communication system. 6. Appropriate relationship between donors and relief teams. Since during such disasters, drugs and medical equipment are donated, there is not much assurance as to their being intact and appropriately preserved. Even, some of them are unsuitable and inapplicable. Hence, those who intend to donate drugs and medical equipment should have a direct relationship with healthcare provider team. 7. Self-management of the patients for chronic diseases should be encouraged and reinforced. 8. There should be an effective communication plan between the individuals and healthcare providers. 9. All the stresses should be controlled (30). 

**Earthquake**

So far, numerous earthquakes have occurred. Out of these earthquakes, the Great East Japan earthquake in 2011 with the magnitude of 9 on Richter scale was one of the most severe ones, which caused triple disasters (38). In addition to its own causalities, it caused tsunami whose casualties were like those of intense flooding. On the other hand, Fukushima Daiichi Nuclear Power Plant was damaged, which caused leakage of radioactive materials (38). Hence, the studies related to this earthquake will be run in two separate parts: The earthquake, and the surrounding area of the Fukushima Daiichi Nuclear Power Plant, which was damaged after the Great East Japan earthquake, is discussed separately. 

Based on the studies performed after the earthquake, prevalence of CVDs such as HTN, ACS, AMI, IHD, HF, VF, sustained or non-sustained VT, and cardiomyopathy and other types of mortality increase after the earthquake (39-79). The rate of CVD outbreak in the regions more impacted and more damaged by the earthquake is higher than other areas. Fatal MI had a significant increase in high impact areas; however, in low impact areas its rate was not different from that of before the earthquake (80). Also, in high impact areas, higher rate of Decompensated HF and AMI is observed, particularly among women and the elderly and those who had to abandon their home (81, 82). 

Impact of the earthquake on CVDs is not permanent and after a period, the incidence rate of CVDs returns to its normal state. The earthquake not only has not had any remarkable impact on long-term prognosis in 30 years, but also has not had any midterm impact on CVDs in 4 years after earthquake in the affected area (83, 84). Some studies indicate that this impact was even less than this and after a few weeks, there was no increase in observed incidence and prevalence of ACS and HTN (43, 47, 51, 59, 76, 85-89). Some studies even express that incidence of CVDs in the first week of the earthquake had a remarkable increase and after that this increase is less observed (43, 90). The less severe earthquakes are, the sooner the return to previous state takes place (40, 85). Conversely, the more severe earthquakes are, the more damage there will be; hence, the increase in incidence of CVDs will last longer and the return to baseline state will occur later; like in Sichuan earthquake with the magnitude of 8 on the Richter scale, where intense destruction occurred and 5 million people were displaced (43, 44, 62, 91, 92). In New Zealand, two earthquakes occurred with an interval of 6 months. The first one was 7.1 on the Richter scale and an increase in CVDs was observed for 3 weeks. However, in the second one with the magnitude of 6.3 on the Richter scale an increase in CVDs was observed for 2 weeks (43). In the less intense earthquakes the rate of CVDs was significantly high only for 3 days; like the two earthquakes that occurred in Thessaloniki, Greece, on 19^th^ and 20^th^ of 1978 with the magnitude of 5.2 and 6.4 on the Richter scale, respectively (93). The other factor impacting the incidence of CVDs is distance from the center of the earthquake. The observed incidence of CVDs such as HTN was lower among those who lived more than 50 km away from the center of the earthquake (51). 

Blood pressure (BP) increases in the people with chronic diseases such as renal failure (94, 95). Other risk factors of increase in BP and uncontrolled BP in the people who live in shelters include being over 55 years old, history of having HTN, and having insomnia. Hence, in addition to taking their previous medication regularly, they probably need to increase their previous medication (83).

The time, at which the earthquake takes place, is another factor affecting the incidence rate of CVDs. For instance, Loma Prieta earthquake, in 1989, took place at 5:04 pm in San Francisco. The magnitude of the earthquake was 7 on the Richter scale. In comparison with the days before or after the earthquake or in comparison with the same day in 1990, on the day of Loma Prieta earthquake, there was not any statistically remarkable increase observed in AMI admission in San Francisco area. Northridge, Los Angeles, earthquake in 1994 occurred at 4:31 am and there was a 110% increase in the rate of AMI admission in Los Angeles on the day of the earthquake in comparison with the mean admission rate over 7 years before the earthquake. Sudden death rate also increased. Therefore, severe emotional stress resulting from sudden wake-up stress affects the increase in AMI. And if there is less stress, AMI risk is lower as well (96, 97). 

In fact, stress plays a pivotal role in increase in incidence of CVDs, which mostly happens because of mental stresses such as losing property and relatives (39, 47, 49, 51, 56, 85, 98). Also, in some studies, white coat is thought to be one of the factors affecting stress and increasing BP after the earthquake (87). In some other studies, signs of depression at the time of admission remarkably predict the risk of re-hospitalization for IHD (44). Mental stress resulting from heavy work leads to increase in the incidence of HTN after the earthquake. Disaster staff, who work in the quaked area, face the risk of increasing HTN if they have a heavy workload (99). Even, ordinary government employees showed a higher rate of increase in HTN in the quaked area. In this study, the average time of monthly extra work of ordinary employees in March, 2011, was 10 times more than public people in the previous March. Therefore, after the earthquake Blood Pressure of government employees should be controlled and if required treatment should be prescribed (75, 100). Also, circadian rhythm changes play a role in increasing fatality resulting from CVDs, which occurred more in the elderly at night and in the morning, but no increase in fatality was observed between 11 am and 11 pm (101). Age, family history of BP, obesity, sleep disorder, waist to hip ratio, high blood sugar, and high-salt food are other factors that affect the increase in incidence of HTN and uncontrolled HTN after the earthquake (47, 62, 101, 102). 

One of the other reasons for uncontrolled Blood Pressure is discontinuity of antihypertensive drugs, which happens because of various reasons. In the people with psychological problems, the risk of stopping using antihypertensive drugs is higher (103). 

One of the cases with different results is a study carried out in New Zealand. In this study, after two earthquakes, there was no increase observed in ventricular arrhythmia (104). Another study expressed that through stimulating sympathetic nerve, earthquake leads to increase in HR and cardiac mortality. However, in the individuals over 60 years of age, stimulation of sympathetic nerve system was blunt (52). In another study, it was said that individuals who lose their residence and live in temporary residence areas, can control their BP as good as the people who live at their own home. However, individuals, particularly the elderly, who live at their own home, indicate increase in BP on winter mornings. Similarly, as to the individuals who do not have any changes in their BP medicine, increase in BP was observed, the researcher did not explain the reasons, though (105). 

**Fukushima Area after the Great East Japan Earthquake**

Areas within 20 km of Fukushima nuclear power plant were determined as high-risk and restricted areas due to nuclear radiations more than 20 mSv per year. Almost all of the residents had to evacuate their homes (106, 107). From 20-30 km of the nuclear plant was determined as area prepared for evacuation at the time of emergency. The areas within 30 km of the plant were determined as deliberate evacuation areas (106).

Incidence of HTN, tachycardia, MI, AF, and the deaths related to CVDs was higher in the individuals who had to abandon their homes (107-113). CVDs risk was higher than normal range within 2 years after the incident (111). However, some studies indicated that incidence of AMI was higher than the surrounding areas only until one month after the incident (107). Other studies held that there was no remarkable difference in the prevalence of AMI before and after the earthquake in Fukushima area (114). 

Stress is a leading factor in increasing the risk of CVDs and hence, a higher rate of CVD is observed in individuals with depression and PTSD (115) and there is a higher increase in the prevalence of CVDs because of psychological stresses like losing property, relatives or job (115, 116). Other risk factors include: previous CVD, being female, being 40-90 years old, obesity, being alcoholic and having dinner late at night (111, 115, 117). After the earthquake, a higher incidence of AF is observed in men compared to other groups (109). 

In comparison between evacuees and non-evacuees, there was no difference or little difference in term of increase in BP (106). 

**Special Groups**

In a study on pregnant women, it was indicated that those who were in their 3^rd^ trimester of pregnancy at the time of incident and stress more commonly had pregnancy HTN (118).

In children under 15years of age, within 1 year of the incident, increase in incidence of HTN is observed (119). It has also been reported that within 4 years of the incident, increase in incidence of HTN in children is observed. In a study on the impacts of great east JAPAN earthquake on the BP of the injured children, it was indicated that the children who went through more stressful incidents like tsunami waves, corpse of their relatives or friends, fire waves or separation from their parents, higher BP was observed. Of these children, those who witnessed fire waves indicated higher diastolic BP (120). 

**Management of CVDs**

One of the important measures to take in order to decrease the risk of CVDs is to strengthen buildings before the earthquake happens. It can be claimed that the less destruction in building, the lower the risk of CVDs (42). 

So as to prevent and treat CVDs, controlling stress is another paramount issue that should be taken into account. Over this period, decrease in stress and coronary risk factors may decrease mortality resulting from Coronary Heart Disease (CHD) after a main EQ (48). Prescription of tranquilizers and anti-depression medication can help control HTN and their prescription may even be essential (121).

After crises, it is more likely that patients stop taking drugs, encouraging hypertensive patients to start taking drugs again may help reduce CVD risk (122). 

After the earthquake, changing the patients’ antihypertensive drugs is another important measure that leads to better control of HTN. Data show that after earthquake, paying special attention to BP level and treatment modifications can be important not only immediately, but also for some months after the earthquake (70). Studies showed that patients who were under treatment of α-blocker or β-blocker or renin-angiotensin inhibitor either did not show any change in their BP level or there was little increase (79).

Supplying a morning home blood pressure measuring device to control morning home blood pressure is essential for preventing CVDs’ side effects (123). Due to many reasons such as losing morning home blood pressure equipment, damage to other equipment, or anxiety caused by vast destruction, most of the patients were not able to measure morning home blood pressure (123). In patients who lived in a shelter, precise control of BP until 4 years was possible using an automatic home sphygmomanometer and web-based information and communications technology (ICT- technology) (124). Controlling BP, nutrition, and personal hygiene can decrease HF as well (102). 

Timely and appropriate intervention is another factor that can help reduce due to CVDs. Complications like HF can occur less under the condition that patients are immediately admitted for AMI and the treatment begins quickly (125). Also immediate admission and intervention improves primary function of PCI (125). 

## Discussion

In this systematic review study, incidence and prevalence of CVDs, morbidity and mortality of CVDs, and treatment and management of CVDs, were investigated. Prevalence of CVDs increases after disasters. This increase directly depends on the intensity of the damage to the disaster area. The most import reason for such an increase are stresses like losing home and relatives and friends, disconnection with friends and relatives, losing job and joblessness, and lack of consistency following the incident. Also, high-risk individuals like the elderly are more susceptible. However, using appropriate medication and non-medication measures in terms of stress, it is possible to decrease prevalence of CVDs. Quick establishment of health clinic and access to appropriate and quick treatment within a few months after disaster is of other measures that can help control CVDs. Such measures like providing and providing access to medication, consulting to change dose or type of medication, and encouraging self-management and self-care can help decrease these complications to minimum. Similarly, it is essential to pay attention to special populations like pregnant women, particularly within their third trimester of pregnancy and children under 15.

In a review study, Kazuomi Kario et al., 2012, studied the effects of 2011 Great East Japan earthquake and Hanshin-Awaji earthquake on CVDs. This study probed into in-clinic and off-clinic HTN, potential mechanism of HTN in disasters, and management of these diseases. The results indicated that BP increases after earthquake and complications related to lifestyle, like stressful factors such as bad quality of sleep, and complications related to activity, such as lack of physical movement after earthquake, can lead to biological rhythm disorders. Aldosterone and cortisone increase in biological rhythm disorders and consequently sympathetic nerve is stimulated, which leads to increase in the use of salt and hence HTN. In this study, controlling use of salt along with establishing a quiet sleeping condition, being away from stress, appropriate physical activity, and having self-management to prevent obesity are mentioned as important factors to control HTN (126). 

Similarly, in our study, controlling stress, encouraging self-care and self-management, and providing BP measuring device to facilitate self-care and self-management are taken to be important factors to control HTN. But, in the study done by Kazumi, providing BP measuring device to facilitate self-care is not mentioned (126). In 2015 and 2016 a guideline titled disaster medicine for CVD was published by Japanese Circulation Society (12). This guideline includes a number of issues like water and food hygiene, salt and sugar regimen, instructions for healthy sleep and providing sound sleep and, if needed, controlling sleeplessness through medication, treating depression, appropriate psychological support, resorting to appropriate diagnostic methods to control CVDs and treat them, and making sure the medication is taken at home to manage and control CVDs (12). Results of this study as well as the clinical guide is consistent with our study, which accentuates controlling stress and providing sound sleep, following a special diet, providing healthy food to the individuals affected by disaster, prescribing medication for sleeplessness, changing HTN medication, controlling risk factors of CVDs, and quickly treating newly-admitted patients or the intensified cases already admitted (12). Results of the study by Errol D et al. are also consistent with our study. That study has also mentioned stress in the disaster area, financial stress resulting from losing job and not having insurance, lack of access to healthy food, salty and high-carbohydrate food, and disconnection with the health system and health service providers as some factors that lead to difficulty in controlling HTN during the hurricane (127). The study has recommended some solutions, like having a list (can be electronic) of medications, presenting data related to complications of not using medication (before disaster), having enough supply of medication, providing appropriate access to medication during disaster, and appropriate control of stress (127). In a systematic review study in 2019, Farzad Gohardehi et al. probed into HTN and diabetes after disasters. Like our study, they indicated that prevalence of HTN remarkably increases after disasters (3). 

**Table 1:** Medline search strategy

**Database**	**Search terms**
MEDLINE (PubMed)	“Cardiovascular Diseases”[Mesh] OR “Pulmonary Edema”[Mesh] OR “Hypertension”[Mesh] OR “Acute Coronary Syndrome”[Mesh] OR “Myocardial Ischemia”[Mesh] OR “Coronary Disease”[Mesh] OR “Congestive heart failure”[Mesh] OR “Coronary Diseases”[tiab] OR “Myocardial Ischemias”[tiab] OR “Ischemic Heart Diseases”[tiab] OR “Ischemic Heart Disease”[tiab] OR “Chronic heart Diseases”[tiab] OR “Chronic heart Diseases”[tiab] OR “Hypertension”[tiab] OR “High Blood Pressure”[tiab] OR “High Blood Pressures”[tiab] OR “Acute Coronary Syndrome”[tiab] OR “Acute Coronary Syndromes”[tiab] OR “Coronary Artery Disease”[tiab] OR “cardiac disease”[tiab] OR “Congestive heart failure”[tiab] OR “Pulmonary Edema”[tiab] OR “Pulmonary Edemas”[tiab] “Myocardial Ischemia”[tiab] OR “Cardiovascular disease”[tiab] OR “Cardiac Disease”[tiab] OR “Cardiac Diseases” OR “Cardiac Disorder”[tiab] “Earthquake”[Mesh] OR ”Floods”[Mesh] OR ”Cyclonic Storms”[Mesh] OR ”Tornadoes”[Mesh] OR ”Natural Disasters”[Mesh] OR ”Disasters”[Mesh] OR “Earthquake” [tiab] OR “Catastrophic Flooding” [tiab] OR “Catastrophic Flooding”[tiab] OR “Floods”[tiab] OR “Cyclonic Storms”[tiab] OR “Cyclonic Storm”[tiab] OR “Cyclone”[tiab] OR “Cyclones”[tiab] OR “Hurricanes”[tiab] OR “Hurricane”[tiab] OR “Tropical Storm”[tiab] OR “Tropical Storms”[tiab] OR “Typhoons”[tiab] OR “Typhoon”[tiab] OR “Tornadoes”[tiab] OR “Tornado”[tiab] OR “Tornados”[tiab] OR “Catastrophic”[tiab] OR “Natural Disasters”[tiab] OR “Natural Disaster”[tiab] 1 &2

**Table 2:** Quality assessment and risk of bias in flood

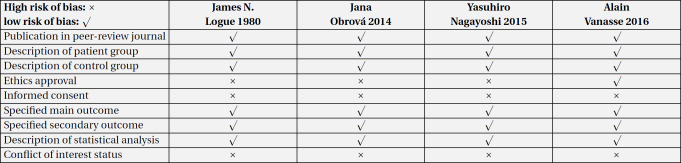

**Table 3:** Quality assessment and risk of bias in storm

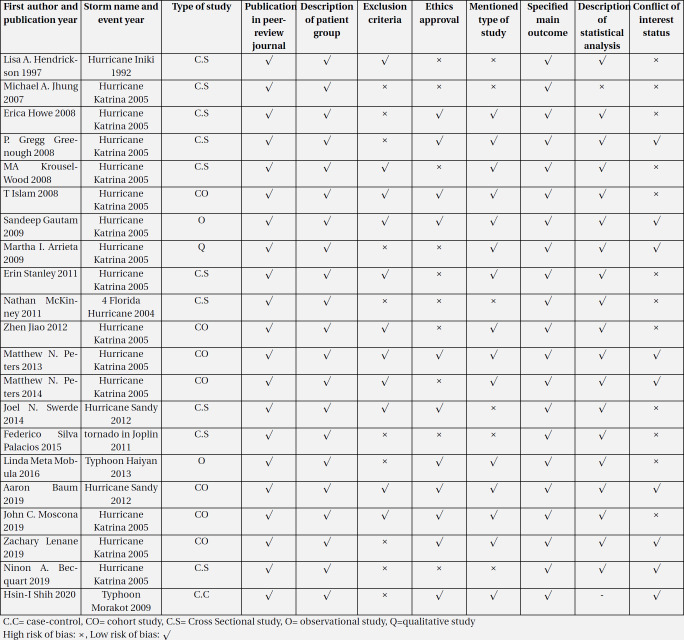

**Table 4:** Quality assessment and risk of bias in earthquake

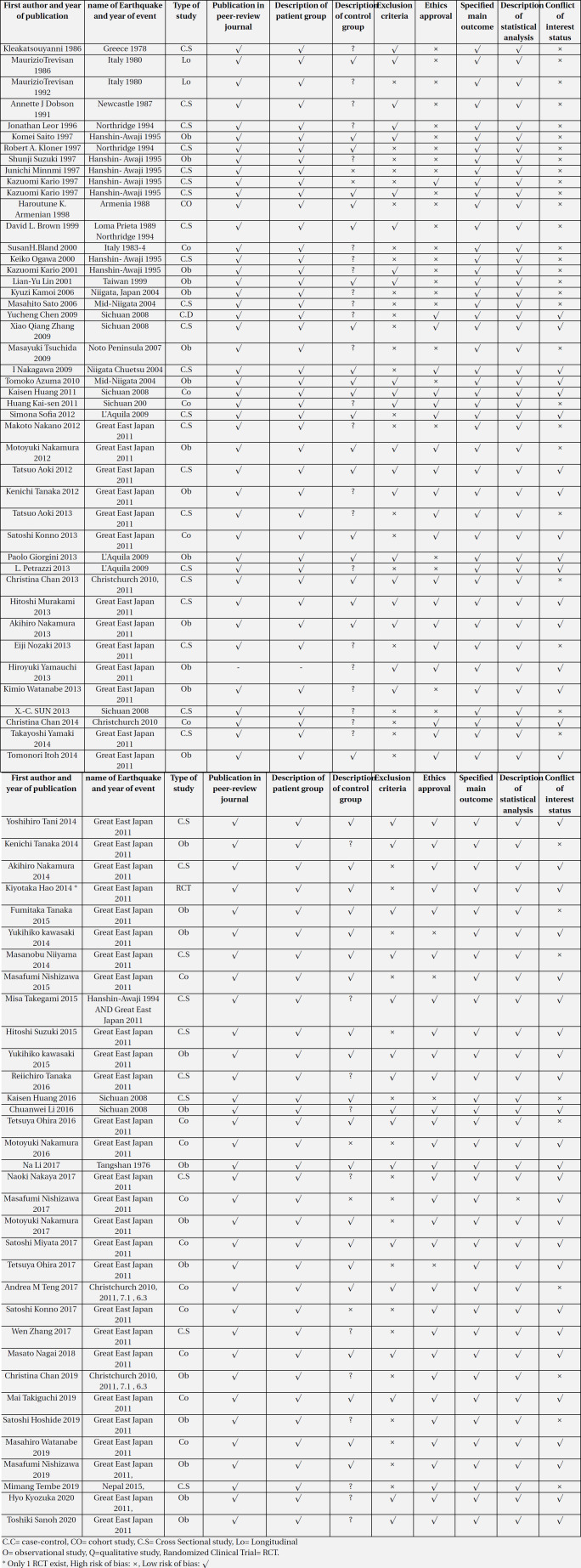

## Limitations

In this study, non-English articles were excluded, which resulted in losing some data. 

## Conclusion:

Prevalence of CVDs increases after disasters. Lack of access to medication or lack of medication adjustment, losing home BP monitor device as a result of destruction, and physical and mental stress after disasters are of the most significant challenges of controlling and managing CVDs. By means of quick establishment of health clinic, providing quick access to appropriate diagnosis and treatment, providing access to medication, and self-management and self-care incentives, along with appropriate medication and non-medication measures to control stress, we can better manage and control cardiovascular diseases, particularly hypertension. 

## Conflict of interest

There is no conflict of interest
